# HIV‐1 Resistance to Dolutegravir in Benin: The Need to Improve and Strengthen Therapeutic Patient Education Strategies to Achieve the Third Objective 95 of UNAIDS 95–95–95 Target

**DOI:** 10.1155/crdi/9324746

**Published:** 2026-06-22

**Authors:** Edmond Tchiakpe, Halimatou Diop-Ndiaye, Sophia Osawe, Coumba Toure-Kane, Akadiri Yessoufou

**Affiliations:** ^1^ Health Ministry, National Reference Laboratory of Health Program Fighting Against AIDS in Benin (LNR/PSLS), Cotonou, Benin, moh.gov.sy; ^2^ Bacteriology and Virology Laboratory Aristide Le Dantec, Cheikh Anta Diop University, Dakar, Senegal, ucad.sn; ^3^ Institute of Human Virology, Abuja, Plateau, Nigeria, ihv.org; ^4^ Institute for Health Research, Epidemiological Surveillance and Training, Cheikh Anta Diop University, Diamniadio Urban Hub, Dakar, Senegal, ucad.sn; ^5^ Laboratory of Cell Biology and Physiology, Abomey-Calavi University, Cotonou, Benin, uac.bj

**Keywords:** antiretroviral treatment, antiretrovirals still effective, Benin, dolutegravir, drug resistance mutations, HIV-1, nonadherence

## Abstract

Benin has adopted the WHO 2019 recommendations by integrating dolutegravir into the first‐ and second‐line treatment regimen with regular monitoring of the emergence of resistance. The standard of care document has been updated, and healthcare staff have been trained on dosage, drug interactions, and the management of adverse effects. The patient, diagnosed as HIV‐1 positive, was started on antiretroviral therapy, which had to be modified due to poor adherence. HIV‐RNA was extracted from plasma with QIAGEN RNA kit, and nested polymerase chain reaction was performed in Reference National Laboratory of Health Program Fighting Against AIDS in Benin on the polymerase gene using the Applied Biosystems HIV‐1 Genotyping Kit PR‐RT with Integrase. Drug resistance mutations and the remaining effective antiretrovirals drug were identified using Stanford University Antiretroviral‐Associated Resistance Mutation Interpretation Algorithm (https://hivdb.stanford.edu/ Version 9.8). The phylogenetic tree was constructed using SeaView with the maximum likelihood method and 1000 bootstrap iterations to identify the viral subtype along PR‐RT and integrase gene. No PI‐associated resistance mutations were observed. The NRTIs and NNRTIs associated resistance mutations were (M41 ML, D67G, S68G, K70R, M184 V, T215F, and K219E), (V179E and Y188 L), respectively. The major and accessory INSTI mutations observed were (E138 K, G140 GA, S147SG, Q148R, and N155H) and T97A, respectively. All protease inhibitors were effective against the identified HIV‐1 viral strain which was a CRF02_AG along the polymerase gene. HIV‐1 resistance genotyping can improve monitoring of the emergence of resistance in nonadherent individuals and anticipate early treatment failures in order to accelerate access to the third 95 of UNAIDS 95–95–95 target.

## 1. Introduction

Antiretroviral treatment (ART) provides inhibition of viral replication. Initially, it consisted of a combination of two nucleoside reverse transcriptase inhibitors (NRTIs) and a non‐nucleoside reverse transcriptase inhibitor (NNRTI) or a protease (PR) inhibitor. However, the emergence of resistance associated with NNRTIs has shown that the latter have a low genetic barrier.

To help countries achieve the UNAIDS 95–95–95 targets by 2030, WHO replaced NNRTIs with dolutegravir in its 2019 recommendations.

In Benin, HIV prevalence is 0.8% [1], and 59,786 people living with HIV (PLHIV) were receiving ART, including 57,656 as first‐line treatment with 57,152 (57,152/57,656; 99.1%) receiving TDF+3 TC + DTG and 504 (504/57,656; 0.9%) receiving ABC+3 TC + DTG by the end of 2024 (statistics are currently available from the PSLS statistics department). By adopting this WHO recommendation, Benin, through its psychologists and educators, could support patients in their therapeutic patient education in order to avoid the emergence of resistance to this antiretroviral which has a strong genetic barrier.

## 2. Case Presentation

The case presented in this manuscript highlights the use of HIV‐1 resistance genotyping and therapeutic patient education sessions to achieve undetectability of HIV‐1 viral load in a patient nonadherent to ART.

This is a man born in 1972 who was referred to a care site for PLHIV in Cotonou in 2019. Each HIV‐1‐infected patient attending the National Reference Laboratory/Health Program Fighting Against AIDS (LNR/PSLS) signs an informed consent authorizing the use of the results of their biological monitoring to improve the health of another patient through studies. Thus, written informed consent was obtained from our patient in this study. The medical records indicated that he had three children but was divorced and the HIV serological test was positive for HIV‐1 on 18/10/2013.

As a reminder, the screening algorithm used was that of two tests: a first sensitivity test “ELISA, Murex Ag⁄Ab Combination assay” and a second specific test “First Response HIV1‐2.0 Card Test (version 2.0) Rapid HIV Card Test (Valsad, Gujarat, India).”

The ART at initiation was d4 T+3 TC + EFV in the medical file. The CD4+ T‐cell value was not recorded. In 2014, d4T was switched to TDF, and the therapeutic regimen was TDF+3 TC + EFV. He remained under this regimen until May 2019, and the CD4+ T‐cell count was 19 cells/mL in November 2018. Therapeutic patient education in May 2019 revealed that the patient reported nonadherence to ART. The viral load was 5.28 log_10_ and the CD4 + T‐cell count was 11 cells/mL. The patient weighed 58 kg. The doctor decided to switch the first‐line to the second‐line AZT+3 TC + LPVr. The events occurring in the patient therapeutic history that led to resistance genotyping are presented in Table 1.

**TABLE 1:** Therapeutic history and events occurring during patient follow‐up in the study.

	**2013**	**2014**	**2018**	**2019**	**2020**	**2021**		**2022**			**2023**	**2024**

**Months**				**May**	**May**	**March**	**December**	**January**	**February**	**November**	**December**	**May**

Therapeutic regimens	d4 T+3 TC + EFV	TDF+3 TC + EFV	TDF+3 TC + EFV	TDF+3 TC + EFV	AZT+3 TC + LPVr	AZT+3 TC + ATVr	AZT+3 TC + ATVr	AZT+3 TC + ATVr	AZT+3 TC + ATVr	AZT+3 TC + DTG	AZT+3 TC + DTG	AZT+3 TC + DTG
Switch of therapeutic regimen	—	—	—	—	AZT+3 TC + ATVr	—	—	—	AZT+3 TC + DTG	—	—	—
CD4+ T‐cell values (cells/mL)	Not recorded	—	19	11	124	—	—	—	—	118	40	—
Adherence to ART	—	—	—	Bad	Bad	—	—	Low	Good	—	—	—
Viral load (Log)	—	—	—	5.28	—	5.71	6.39			2.99	5.6	4.75
Weight (kilograms)	—	—	—	58	63	59	—	—	—	—	62	53
Adverse effects	—	—	—	—	—	—	—	—	Related to ATVr	—	—	—
Events	—	—	—	—	LPVr shortage	—	—	—	—	—	—	—
Therapeutic patient educations	—	—	—	Yes	Yes	—	—	Yes	Yes	—	—	—
Doctor’s request	—	—	—	—	—	—	—	—	—	—	—	Resistance genotyping

*Note:* ART, antiretroviral treatment; LPV, lopinavir; ABC, abacavir; AZT, zidovudine; FTC, entricitabine; 3 TC, lamivudine; TDF, tenofovir; DTG, dolutegravir; r, ritonavir; d4T, stavudine.

Abbreviation: CD: cluster of differentiation.

The physician requested genotyping for antiretroviral resistance. Blood samples were collected and separated into two aliquots. One was used for viral load, and the second was divided into two (BEN8981) and (BEN8982) for genotyping of PR and reverse transcriptase (RT) and integrase (IN) genes, respectively. The viral load was carried out using Cobas 5800 System and yielded a value of 4.75 log_10_ on 06/05/2024.

HIV‐RNA was extracted from plasma with QIAGEN RNA kit, and nested polymerase chain reaction (PCR) was performed in LNR/PSLS on the polymerase gene using the Applied Biosystems HIV‐1 Genotyping Kit PR‐RT with IN.

The DNA sequences obtained by PCR were cold precipitated then purified and sequenced. The sequences were edited using the DNASTAR SeqMan Pro and directly submitted to the Stanford University Antiretroviral‐Associated Resistance Mutation Interpretation Algorithm (https://hivdb.stanford.edu/Version 9.8) to identify the mutation positions.

The phylogenetic tree was constructed using SeaView with the maximum likelihood method and 1000 bootstrap iterations to characterize the viral subtype along PR‐RT and IN gene.

No PI‐associated resistance mutations were observed. The NRTIs and NNRTIs resistance mutations were (M41 ML, D67G, S68G, K70R, M184 V, T215F, and K219E) and (V179E and Y188 L), respectively. The major and accessory INSTI mutations observed were (E138 K, G140 GA, S147SG, Q148R, and N155H) and T97A, respectively. The remaining effective drugs identified are presented in Table 2.

**TABLE 2:** Resistance mutations associated with protease, reverse transcriptase and integrase, and still effective antiretrovirals identified on the HIV‐1 virus carried by the patient.

		**Acid amino substitution positions**	**Sensitivity or resistance of antiretroviral drugs**	
			**Sensitive**	**Resistant**

Interpretation of antiretroviral resistance mutations: BEN 8981	Major mutations associated with PIs	None	ATV/*r*, DRV/*r*, LPV/*r*	None
	Accessory mutations associated with PIs			
	Major mutations associated with NRTIs	M41 ML, D67G, S68G, K70R, M184 V, T215F, K219E	None	ABC (HLR), AZT (HLR), FTC (HLR), 3 TC (HLR), TDF (IR)
	Major mutations associated with NNRTIs	V179E, Y188L	None	DOR (HLR), EFV (HLR), ETR (LLR), NVP (HLR), RPV (HLR)

Interpretation of antiretroviral resistance mutations: BEN 8982	Major mutations associated with INSTIs	E138 K, G140 GA, S147SG, Q148R, N155H	None	BIC (HLR), CAB (HLR), DTG (HLR), EVG (HLR), RAL (HLR)
	Accessory mutations associated with INSTIs	T97A	None	

*Note:* ATV: atazanavir, DRV: darunavir, LPV: lopinavir, PR: protease, IN: integrase, ABC: abacavir, AZT: zidovudine, FTC: entricitabine, 3 TC: lamivudine, TDF: tenofovir, DOR: doravirine, EFV: efavirenz, ETR: etravirine, NVP: nevirapine, RPV: rilpivirine, BIC: bictegravir, CAB: cabotegravir, DTG: dolutegravir, EVG: elvitegravir, RAL: raltegravir. D: aspartic acid, F: phenylalanine, E: glutamic acid, Y: tyrosine, K: lysine, R: arginine, Q: glutamine, N: asparagine, BEN: Benin.

Abbreviations: A, alanine; G, glycine; H, histidine; HLR, high‐level resistance; IR, intermediate resistance; L, leucine; LLR, low‐level resistance; M, methionine; NNRTI, non‐nucleoside reverse transcriptase inhibitors; NRTI, nucleoside reverse transcriptase inhibitors; PI, protease inhibitors; RT, reverse transcriptase; S, serine; T, threonine; V, valine.

The HIV‐1 strain identified was CRF02_AG along the polymerase gene. The new sequences have been deposited in GenBank under the accession numbers PQ115151 and PQ115152.

## 3. Discussion

Reviewing the patient’s medical history, we expected an increase in viral load after the treatment with TDF+3 TC + DTG, especially since there were recurrent episodes of nonadherence to ART. In our study, the viral load was 4.75Log copies/mL, and the persistence of viral load has been reported among patients with DTG in Brazil [2] and Lesotho [3]. We must quickly become aware of this situation and avoid finding ourselves in situations where resistance to NNRTIs has been reported in patients with or without ART [4]. Despite its strong genetic barrier [5], HIV‐1 rapidly selects mutations that confer resistance to DTG in the event of treatment nonadherence. In this study, we observed INSTIs major (E138 K, G140 GA, S147SG, Q148R, and N155H) and accessory (T97A) mutations. These mutations are known to reduce the sensitivity of HIV‐1 to DTG, as reported by studies conducted in Tanzania [6] and Turkey [7]. The mutations (Q148R and N155H) reduce the effectiveness of INSTIs and are often associated with secondary mutations to confer increased resistance and improve the replicative capacity of the virus through two pathways: The Q148R pathway with mutations at Positions 140 and 138, and the N155H pathway linked to mutations at Positions 140 and 97 [8].

The presence of the Q148R and N155H mutations suggests that the virus carried by our patient was exposed to either raltegravir or elvitegravir. Indeed, the selection of these two mutations by the virus improves viral replication in patients who have failed raltegravir‐ or elvitegravir‐based ART [9]. In Benin, raltegravir was included in the third‐line treatment regimen for patients who had failed both first‐ and second‐line therapy [10, 11]. These two mutations have been reported in patients who have failed treatment with raltegravir‐based ART in the United States [12] but also in Paris in France [13] and sub‐Saharan Africa [14].

Currently, fusion inhibitors are not available in the country, and the patient was unwilling to pay for this expensive treatment [15].

On this basis, the patient’s file was studied by a third‐line committee [16], which offered him a line of treatment based on drugs that were still remaining effective against the virus. Currently, the patient is undergoing dual therapy comprising TDF and LPV/*r* (Table 2). The patient was advised to strengthen therapeutic adherence, and a viral load test was performed after 3 months and gave HIV‐1 target not detected result.

CRF02_AG was the predominant HIV‐1 strain isolated in all studies conducted in Benin [17, 18] and the West African subregion [19, 20]. Its characterization in this study indicates that it continues to circulate within and outside the borders of Benin.

Benin has benefited from USAID support through the PEPFAR project to train teams to care for PLHIV in the various treatment centers in Benin. Peer educators and psychologists who have received this training help PLHIV to identify the reasons for their nonadherence to daily medication treatment. One of the mechanisms implemented in Benin that improves care is the referral of patients with a viral load greater than 3log_10_ to mediators and psychologists responsible for calling them for therapeutic education appointments once the viral load results are available from the laboratory and have reached the treatment centers. The costs of these phone calls are included in the milestones assigned to each center that has reached the defined performance level. In addition to the Applied Biosystems 3500 DNA sequence in the country, the supply of QIAGEN RNA extraction kit and Applied Biosystems HIV‐1 Genotyping Kit PR/RT with IN sequencing is continuous to offer routine genotyping to PLHIV‐1.

Our study is not without limitations. We do not have complete data on the patient therapeutic history. Indeed, the patient could be exposed to raltegravir, used as a third‐line treatment in the country, and not declared it during therapeutic patient education or the healthcare staff who treated the patient may have forgotten to document all the administered treatment regimens in the care record.

## 4. Conclusion

Our case highlights the consequences of poor adherence to DTG‐based ART. HIV‐1 resistance genotyping can improve surveillance of the emergence of resistance mutations in nonadherent individuals, thereby enabling the anticipation of early treatment failures in order to accelerate access to the third target 95 of UNAIDS 95–95–95 goal.

**Nomenclature**
BEN: Benin
DTG: Dolutegravir
HIV: Human immunodeficiency virus
LNR/PSLS: National Reference Laboratory/Health Program Fighting Against AIDS
ELISA: Enzyme‐linked immunosorbent assay
UNAIDS: United Nations Programme on HIV/AIDS
CD4: Cluster de différenciation 4
D4 T: Stavudine
TDF: Tenofovir
3 TC: Lamivudine
EFV: Efavirenz
AZT: Zidovudine
LPVr: Lopinavir ritonavir
ATVr: Atazanavir ritonavir
PR: Protease
RT: Reverse transcriptase
RNA: Ribonucleic acid
DNA: Deoxyribonucleic acid
PCR: Polymerase chain reaction
M: Methionine
D: Aspartic acid
G: Glycine
S: Serine
K: Lysine
R: Arginine
V: Valine
T: Threonine
F: Phenylalanine
E: Glutamic Acid
Y: Tyrosine
L: Leucine
A: Alanine
Q: Glutamine
N: Asparagine
H: Histidine

## Author Contributions

Edmond Tchiakpe and Halimatou Diop‐Ndiaye conceptualized the study and drafted the initial manuscript. Sophia Osawe, Coumba Toure‐Kane, and Akadiri Yessoufou revised the manuscript and approved the final version.

## Funding

This research did not receive any specific grant from funding agencies in the public, commercial, or not for profit sectors.

## Disclosure

The present manuscript represents the version submitted for formal peer review and consideration for publication and has not been previously published in a peer‐reviewed journal. All authors reviewed and approved the final manuscript. This research presents an accurate account of the work performed, all data presented are accurate, and methodologies detailed enough to permit others to replicate the work. All authors have been personally and actively involved in substantive work leading to the manuscript and will hold themselves jointly and individually responsible for its content.

## Ethics Statement

Edmond Tchiakpe testifies on behalf of all coauthors that their submitted article followed ethical principles in publishing.

## Consent

Written informed consent was obtained from the patient for the publication of this case report.

## Conflicts of Interest

The authors declare no conflicts of interest.

## Data Availability

The data supporting the findings of this study are included within the manuscript.

## References

[1] UNAIDS , UNAIDS.Org/En/Resources/Documents_Unaids_Data, 2022, UNAIDS.

[2] Diaz R. S. , Hunter J. R. , Camargo M. et al., Dolutegravir-Associated Resistance Mutations After First-Line Treatment Failure in Brazil, BMC Infectious Diseases. (2023) 23, no. 1, 10.1186/s12879-023-08288-8. PMC10210384 37226112

[3] Tschumi N. , Lukau B. , Tlali K. et al., Emergence of Acquired Dolutegravir Resistance in Treatment-Experienced People With HIV in Lesotho, Clinical Infectious Diseases. (2024) 79, no. 5, ciae185–1222, 10.1093/cid/ciae185. PMC11581688 38567806

[4] Musengimana G. , Tuyishime E. , Kiromera A. et al., Acquired HIV Drug Resistance Among Adults Living With HIV Receiving First-Line Antiretroviral Therapy in Rwanda: A Cross-Sectional Nationally Representative Survey, Antiviral Therapy. (2022) 27, no. 3, 10.1177/13596535221102690. PMC9263597 35593031

[5] Yoshinaga T. , Miki S. , Kawauchi-Miki S. , Seki T. , and Fujiwara T. , Barrier to Resistance of Dolutegravir in Two-Drug Combinations, Antimicrobial Agents and Chemotherapy. (2019) 63, no. 3, 10.1128/AAC.02104-18. PMC6395897 30602514

[6] Bwire G. M. , Aiko B. G. , Mosha I. H. et al., High Viral Suppression and Detection of Dolutegravir-Resistance Associated Mutations in Treatment-Experienced Tanzanian Adults Living With HIV-1 in Dar Es Salaam, Scientific Reports Nature Publishing Group. (2023) 13, no. 1, 10.1038/s41598-023-47795-1. PMC10665358 37993493

[7] Sayan M. , Yildirim F. S. , Akhan S. , Karaoglan I. , and Akalin H. , Integrase Strand Transfer Inhibitor (INSTI) Genotypic Resistance Analysis in Treatment-Naive, INSTI Free Antiretroviral-Experienced and INSTI-Experienced Turkish Patients Infected With HIV-1, Current HIV Research. (2022) 20, no. 2, 184–192, 10.2174/1570162X20666220303104509. 35240975

[8] Foka F. E. T. and Mufhandu H. T. , Current ARTs, Virologic Failure, and Implications for AIDS Management: A Systematic Review, Viruses. (2023) 15, no. 8, 10.3390/v15081732. PMC10458198 37632074

[9] Andreatta K. N. , Miller M. D. , and White K. L. , Drug Susceptibility and Viral Fitness of HIV-1 With Integrase Strand Transfer Inhibitor Resistance Substitution Q148R or N155H in Combination With Nucleoside/Nucleotide Reverse Transcriptase Inhibitor Resistance Substitutions, Antimicrobial Agents and Chemotherapy. (2016) 60, no. 2, 757–765, 10.1128/AAC.02096-15. 26574015 PMC4750659

[10] PNLS and Politique , Normes et Procédures Pour La Prise En Charge Des Personnes Vivant Avec Le VIH, 2012, https://www.medbox.org.

[11] Tchiakpe E. , Keke R. K. , Diouara A. A. M. et al., Prevalence of Pretreatment HIV-1 Resistance to Integrase Strand Transfer Inhibitors in Newly Diagnosed and Antiretroviral Therapy-Naive Adults in Benin, West Africa, Journal of Global Antimicrobial Resistance. (2026) 46, 112–116, 10.1016/j.jgar.2025.11.011. 41265638

[12] Fransen S. , Gupta S. , Danovich R. et al., Loss of Raltegravir Susceptibility by Human Immunodeficiency Virus Type 1 is Conferred via Multiple Nonoverlapping Genetic Pathways, Journal of Virology. (2009) 83, no. 22, 11440–11446, 10.1128/JVI.01168-09. 19759152 PMC2772690

[13] Charpentier C. , Karmochkine M. , Laureillard D. et al., Drug Resistance Profiles for the HIV Integrase Gene in Patients Failing Raltegravir Salvage Therapy, HIV Medicine. (2008) 9, 765–770, 10.1111/j.1468-1293.2008.00628.x. 18651855

[14] Semengue E. N. J. , Santoro M. M. , Ndze V. N. et al., HIV-1 Integrase Resistance Associated Mutations and the Use of Dolutegravir in Sub-Saharan Africa: A Systematic Review and Meta-Analysis, PLOS Global Public Health Public Library of Science. (2022) 2, 10.1371/journal.pgph.0000826. PMC10021461 36962573

[15] Jamjian M. C. and McNicholl I. R. , Enfuvirtide: First Fusion Inhibitor for Treatment of HIV Infection, American Journal of Health System Pharmacy. (2004) 61, no. 12, 1242–1247, 10.1093/ajhp/61.12.1242. 15259753

[16] MS/PSLS/BENIN , Politique, Normes Et Procédures De Prise En Charge Des Personnes Vivant Avec Le VIH Au Bénin, 2019, Medbox.

[17] Fofana D. B. , Diarra H. , Guindo I. et al., Prevalence of HIV-1 Natural Polymorphisms and Integrase-Resistance-Associated Mutations in African Children, Viruses. (2023) 15, no. 2, 10.3390/v15020546. PMC9964382 36851760

[18] Monleau M. , Faihun F. , Affolabi D. et al., Antiretroviral Drug Resistance in HIV-1 Infected Patients Receiving Antiretroviral Treatment in Routine Clinics in Cotonou, Benin, Journal of AIDS and HIV Research. (2011) 6, 114–120.

[19] Farinre O. , Gounder K. , Reddy T. et al., Subtype-Specific Differences in Gag-Protease Replication Capacity of HIV-1 Isolates From East and West Africa, Retrovirology. (2021) 18, no. 1, 10.1186/s12977-021-00554-4. PMC8097975 33952315

[20] Li R. , Gao Y. , Song T. et al., Global and Regional Molecular Epidemiology of HIV-1 Among Men Who Have Sex With Men: A Systematic Review and Meta-Analysis, AIDS Research and Therapy. (2025) 22, no. 1, 10.1186/s12981-025-00776-y. PMC12398045 40886010

